# Models of teaching medical errors

**DOI:** 10.12669/pjms.37.7.4506

**Published:** 2021

**Authors:** Gassem Gohal

**Affiliations:** 1 Dr. Gassem Gohal, MD, FRCPC, ABP. Department of Pediatrics, Jazan University, Faculty of Medicine, Jazan, Saudi Arabia

**Keywords:** Medical errors, Model of teaching, disclosure, Patient Safety, Adverse events

## Abstract

Medical errors are relatively common causes of preventable iatrogenic adverse events. We have focused on teaching models in certain courses of study that have been reported to have significant positive impacts on the outcomes of teaching about medical errors. All healthcare organizations must establish suitable models of teaching about patient safety and medical errors as a preventive measure and as an early intervention strategy. Teaching undergraduate medical students and physicians in training how to manage and disclose medical errors helps them develop lifelong skills that can effectively reduce such errors.

The literature search was conducted in international databases such as PubMed/MEDLINE and Google Scholar search engine using English equivalent keywords, from 1998 up to April, 2020. The search strategy used the following subject headings terms: “Medical error(s)” AND “Teaching”. Out of 40 Studies included, 6 studies were selected to have evaluated models of health care training and simulation based teaching of medical errors and patient safety in undergraduate and postgraduate medical education.

## INTRODUCTION

A medical error is defined as a preventable adverse event that is the result of a failure in medical care.^1^ The catastrophic effect of medical errors can result in patient death or adversely affect patient quality of life. The burden of medical errors not only affects patients but also can impact physician health, well-being, and performance through increased emotional distress, fear of practicing, increased caution, worsening of doctor-patient relations, and the loss of societal trust in the health care system and physicians.^2^

Medical errors are the third leading cause of death in the USA.^3^ In Australia, medical errors result in as many as 18 000 unnecessary deaths annually.^4^ In Canadian reports, adverse medical events occur in 7.5% of hospital admissions annually, and close to 70,000 of those adverse events were potentially preventable.^5^

Errors in medical practice primarily occur in health care systems that fail to recognize the risk of medical errors and do not take steps to prevent them.^6^ Medical errors can occur as a result of unsafe individual human behaviors, such as prescribing medication or performing procedures in the absence of the necessary levels of knowledge and experience.^7^ Errors can also be the result of working under stressful conditions, such as sleep deprivation, which has a significant negative effect on medical staff, physicians and nurses.^8^,^9^ resulting in a higher risk of medical errors. Certain patient populations have an elevated risk of experiencing medical errors, such as children, who are at especially high risk for medication errors,^10^ and elderly patients, who often have multiple comorbidities.^11^ The risk of errors is higher in certain medical and surgical disciplines, such as cardiothoracic surgery, vascular surgery, neurosurgery, pediatric intensive care, and emergency care.^4^,^12^

Medical errors can be categorized, according to the stage of medical intervention, as diagnosis and management. Errors related to diagnosis include, missed diagnoses, delayed diagnoses or misdiagnoses.^6^ Errors related to management include, but are not limited to, using the wrong parameter to guide treatment, prescribing the wrong dose or medication, performing a surgical operation at the wrong site, delivering medication to or operating on the wrong patient.^13^-^15^ One fundamental, well-validated factor that is responsible for medical errors is poor communication between medical professionals.^16^-^18^

There is increasing desire on the part of health care societies to integrate medical error and patient safety education into their curricula and training programs.^19^ The main aim is to improve the quality of health care through the early introduction of topics pertaining to patient safety and medical error. To the best of our knowledge, there are no policies mandating the teaching of medical errors in medical schools worldwide.

Our goal in this review is to focus on selected models for teaching about medical errors in undergraduate medical schools and physician training programs for which there is strong evidence of a positive effect to support the establishment of similar models in our local medical schools and health care institutions and to make this information widely available.

## METHODS

This review was designed to include studies with model based medical education regarding medical errors and patient safety. Searches were completed in three major databases, including PubMed/MEDLINE and Google Scholar, from 1998 up to April 2020, Search was restricted to the English language. Studies were included if they had simulations or feedback and assessment-based education models. References from the selected studies and from other relevant articles were screened for potential additional studies. The aim of this review was to explore the modalities of teaching medical errors with a high positive impact on healthcare professionals in undergraduate and postgraduate.

## RESULTS

A total of 2340 studies were screened from MeSH-Pubmed/MEDLINE and Google Scholar. Total 40 studies were included for the purpose of this review and six selected studies matched our requirements. These studies included designed training courses or web-based education programs with structured feedback to measure the positive impact on students, physicians and other healthcare professionals. We reviewed those six studies comprehensively and have described each of their models for teaching medical errors, in order to build similar models in medical school and healthcare facilities.

### Model-1

It was characterized by the early integration of medical error education into the curriculum for third year medical students in the form of a four-hour course that included interactive discussions, readings, and a videotaped session with a standardized patient. Each student had a ten-to-fifteen-minute videotaped encounter with a standardized patient. The student was asked to use basic interviewing skills, discuss the error, practice apologizing for the error, take responsibility for the error, admit they did not know something, and attempt to reestablish the patient’s trust. This encounter was followed by a small-group feedback session that lasted for two hours and included the participating students, the standardized patients, a family medicine physician, and a behavioral medicine faculty member. The course was evaluated based on pre and post course questionnaires. Most students were successful at honestly disclosing the error, taking responsibility, and apologizing for the error. Many students felt relieved after participating in these difficult encounters and the authors of this study believed that this model gave students valuable experience that increased their awareness of patient safety and medical errors.^19^

### Model-2

Morbidity and mortality (M&M) meetings are forums in which adverse outcomes are discussed.^20^ M&M meetings are considered powerful tools to improve the quality of care by discussing patients who have experienced adverse effects as a result of medical care.^21^

One interactive, collaborative M&M model was characterized by a 1-hour monthly workshop in which a case of mortality was reviewed by a small group that included physicians, nurses, administrators, pharmacists, and patient safety officers. The chief resident presented a structured timeline of the case, and faculty members facilitated the discussion among residents as follows:

- The PGY-1 group was assigned to analyze the error and determine whether the medical error caused the adverse event.
- The PGY-2 group was tasked with conducting a root cause analysis (RCA) with a fishbone diagram and to generate an action plan based on the RCA.
- The PGY-3 group was charged with determining if the standard of care was met and to rate the effectiveness of the PGY-2 action plan.

The authors of this interactive M&M model observed the excitement and motivation of trainees with regard to learning about patient safety and establishing lifelong habits that could effectively reduce medical errors.^22^

### Model-3

A storytelling teaching session was conducted using video animations of medical cases involving patient harm as described by junior physicians. After each animated story was played, two faculty members facilitated a large-group discussion. A fishbone analysis tool was used to guide the identification of the factors that contributed to each case, and the post course evaluation showed that the session was an effective method of teaching about patient safety and increasing awareness of medical errors. The unique aspects of this model are the power of the emotional influence of storytelling in clinical practice and the use of those stories to transfer experience and improve clinical skills.^23^,^24^

### Model-4

Simulation-based training courses in surgical and medical training programs are popular models for teaching about patient safety and medical errors.^25^ A good example is a simulation-based curriculum presented by Riefkohl-Ortiz et al. during which emergency medicine trainees learned how to manage iatrogenic critical care procedure complications. Trainees were tasked with engaging in six simulated emergency clinical scenarios. The trainees were encouraged to make errors, which led to iatrogenic complications in the high-fidelity patient simulators. Then, the trainees were asked to reflect on how the errors or mismanagement impacted their understanding of the scenario.

Encouragement, the ability to make errors without the risk of harming patients, the repetition of the scenarios followed by debriefing, and the provision of direct supervision with hands-on bedside training were the novel characteristics of this model.^26^

This curriculum lasted for three days. On Day-1, learners were told to manage each simulated case to the best of their ability to assess their baseline performance, and each learner completed an assessment of their confidence regarding the management of iatrogenic injuries. On Day-2, the educational intervention was performed. The trainees engaged in six 10-minute simulation scenarios as a group with a subsequent faculty-led bedside debriefing and didactic lecture lasting 45 to 50 minutes (6 hours total). On Day-3, the trainees completed the same confidence assessment surveys a second time. Using simulations to teach about medical errors and the management of related complications is a useful strategy for reducing the adverse effects of medical errors care and improving the outcomes.^26^


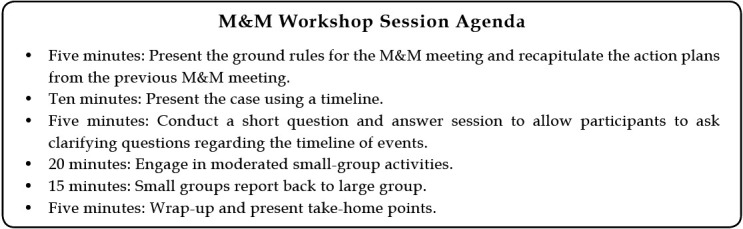


### Model-5

The Agency for Healthcare Research and Quality (AHRQ) has developed an M&M website that presents cases of confidentially reported medical errors that are accompanied by expert commentaries supported by evidence. Five cases are posted each month to address diverse patient safety issues. Providers who submitted their cases of medical errors felt positive about their submission because the system ensured confidentiality, and a small honorarium was paid to those whose cases were accepted for publication. The AHRQ Web M&M is considered one of the most successful experiments in patient safety reporting and medical error education.^27^ Such a web-based secured forum is an ideal solution for large health care organizations seeking to design uniform educational and interventional programs for their staff that would enable them to report and learn about patient safety and medical error issues.

### Model-6

Physicians often express that clinical mistakes have a significant negative affect on their self-esteem.^28^ Fear of legal action in the form of malpractice suits may contribute to the failure of medical professionals to fully disclose some medical errors.^29^ Some models of how to teach medical error disclosure have been evaluated.^30^ We found that the modified communication tool for giving negative news (e.g., the SPIKES mnemonic) used by Barrios, L, et al, in their study is a useful practical tool that can be used to develop a model for medical error education.^31^ The educational experience of junior and senior surgical trainees was augmented with simulated clinical scenarios of iatrogenic injuries that were videotaped and evaluated by two reviewers using a modified SPIKES protocol.

SPIKES, a validated tool for the delivery of negative news, includes the following parameters:

1. Setting up the interview.
2. Assessing the patient’s perception.
3. Obtaining the patient’s invitation.
4. Providing knowledge and information to the patient.
5. Addressing the patient’s emotions with empathetic responses.
6. Providing a strategy and summary.^31^

This six-step strategy was evaluated based on a Likert scale, where;

1 = Poor, 2 = Fair, 3 = Good, 4 = Very Good, and 5 = Excellent.

The authors of this study found this model, including the simulations and evaluations based on the modified SPIKES protocol, useful for teaching trainees about the disclosure of iatrogenic injuries.

## DISCUSSION

In this review, we aimed to review studies presenting structured models of teaching medical errors. We found that there are a limited number of studies in the literature focusing on teaching about medical error.^32^,^33^ Most of the reported studies of medical errors focus on the psychological effect of medical errors among healthcare workers.^34^ Healthcare workers’ level of competency was found to be associated with their level of awareness of and attitude toward medical error.^35^ For this reason, we believe in using a formative, structured way of teaching about medical error to healthcare professionals, even during early clinical encounters in medical school and training programs. Some of the discussed models could be piloted to develop suitable and effective strategies to teach medical errors.^36^ The disclosure of medical errors was shown to prompt tension in communication among health care providers.^37^ The use of simulation-based education showed the highest positive evidence to improve the ability to disclose medical errors.^38^,^39^ Simulation based education, is a preferable method to teach and train healthcare professionals, improve their skill and attitudes while protecting trainees and patients from unnecessary burdens and risks respectively. We preferred the models that used simulation in teaching recognition, disclosure, and adverse effects of medical errors and promotion of patient safety.^40^

Based on this review, we encourage medical schools and health care organizations to use and evaluate some of these suggested models to help grow local experience and encourage the sharing of experiences to promote patient safety by ensuring early intervention strategy. We advise continually updating these models to cope with current and emerging medical situations, like the global pandemic of COVID-19, which may carry potential risks for medical error, especially given higher levels of stress and precautionary procedures.^41^

### Limitations of the study

This review is not a meta-analysis for all studies of medical error. There are several limitations in our review related to the low number of studies concerning the teaching of medical error. Indeed, most of these reported studies are not recent i.e., within the past five years. Furthermore, some of the articles only use small sample sizes. Other limitations include heterogeneity and variability between studies.

## CONCLUSION

Medical errors will be encountered persistently as it is part of human nature to make mistakes. However, it is important to develop methods of reducing medical errors. Certain models for teaching students and physicians about medical errors and patient safety are documented in the literature. Each health care system should develop rigorous models to teach professionals how to recognize, evaluate and disclose medical errors so as to ensure patient safety

### Author’s Contribution:

**GG** did the study design, data collection, manuscript writing, editing, revision, approved final manuscript, and was responsible for the integrity of research.
